# 淋巴浆细胞淋巴瘤/华氏巨球蛋白血症诊断与治疗中国指南（2022年版）解读

**DOI:** 10.3760/cma.j.issn.0253-2727.2022.12.003

**Published:** 2022-12

**Authors:** 文婕 熊, 树华 易, 录贵 邱

淋巴浆细胞淋巴瘤/华氏巨球蛋白血症（lymphoplasmacytic lymphoma/Waldenström macroglobulinemia，LPL/WM）是一种少见的惰性成熟B细胞淋巴瘤。近年，随着对其发病机制探索的深入，以及治疗新药的涌现，广大医务人员对其的关注度普遍提高。2016年由中国抗癌协会血液肿瘤专业委员会牵头制定的中国首个LPL/WM诊断与治疗中国专家共识进一步规范了LPL/WM诊疗标准，对提高我国临床工作者对LPL/WM的认识起着重要的作用。近期，中国抗癌症协会血液肿瘤专业委员会组织全国相关专家讨论并制定了2022版LPL/WM中国指南，针对LPL/WM诊疗中比较关注但指南中未能展开讨论的几个问题，我们在此进行解读，以便更好地指导医务工作者使用本指南。

一、LPL与WM的关系

LPL/WM定义是由小B淋巴细胞、浆样淋巴细胞和浆细胞组成的淋巴瘤，常常侵犯骨髓，也可侵犯淋巴结和脾脏，并且不符合其他可能伴浆细胞分化的小B细胞淋巴瘤诊断标准[1]–[2]。LPL侵犯骨髓伴血清单克隆性IgM丙种球蛋白时诊断为WM。90％～95％的LPL为WM，仅小部分LPL患者分泌单克隆IgA、IgG或不分泌单克隆性免疫球蛋白。因此，WM是LPL主要的疾病类型，且必须同时存在骨髓侵犯及单克隆性IgM丙种球蛋白。若为除IgM以外其他类型免疫球蛋白升高则诊断为LPL，而不能诊断为WM。另外，还有极少数患者存在双克隆或为非分泌型，但双克隆患者比例极低，多数为病例报道，预后情况尚不明确。中国医学科学院血液病医院系统分析了340例LPL患者，其中非IgM的患者23例（6.8％），分泌单克隆性IgG 14例（4.1％，9例为κ轻链，5例为λ轻链），不分泌单克隆性免疫球蛋白7例，分泌单克隆性IgA 2例（0.6％，均为κ轻链）[3]。近期另一项瑞典淋巴瘤中心登记研究纳入1 511例LPL/WM患者，其中仅33例患者为非IgM型LPL（54％为IgG，15％为IgA），12％为非分泌型。与WM患者相比，非IgM型LPL患者更年轻，诊断时有更多的不良预后因素，如LDH升高、贫血和淋巴细胞增多等。此外，非IgM型LPL患者更易出现症状并在诊断时需要接受治疗。但非IgM型LPL和WM患者总生存（OS）的差异并无统计学意义[4]。

二、WM诊断中的难点问题

WM虽然有诊断标准（见指南）[5]，但因每一条标准难以标准化，因此给临床诊断带来很多不确定性，临床诊疗中仍存在许多难点：

1. IgM定量数值差异较大。WM目前定义为LPL侵犯骨髓伴血清单克隆性IgM丙种球蛋白，但并未对IgM具体数值进行要求。虽然多数患者存在IgM升高，但仍有部分患者IgM定量检测正常或轻度升高。因此对于IgM正常或轻度升高的患者，若未进行免疫固定电泳检测，容易出现漏诊及误诊。此外，IgM升高并非WM特异性指标，多种B细胞慢性淋巴增殖性疾病（B-LPD）可伴有血清单克隆性IgM成分[6]，并出现浆细胞分化的形态学特征，从而较难与WM鉴别，如慢性淋巴细胞白血病/小细胞淋巴瘤、套细胞淋巴瘤、边缘区淋巴瘤、滤泡性淋巴瘤等。

2. 典型LPL/WM存在两至三群可识别的细胞形态，即成熟小淋巴细胞、浆细胞、淋巴样浆细胞，但该特点不是LPL特有，且形态诊断一致性差，无法进行标准化。近年来，应用流式细胞术同时检测单克隆B淋巴细胞和单克隆浆细胞成为LPL/WM诊断的重要检查，但同时存在单克隆B细胞和单克隆浆细胞并非LPL/WM特征性表现，且不是所有LPL/WM均存在流式细胞术可检测的两群单克隆细胞。

3. WM侵犯骨髓的典型表现为小梁间隙浸润模式，与脾边缘区的窦内侵犯和滤泡性淋巴瘤的小梁旁侵犯模式不同，这些骨髓病理特点也是WM与其他B细胞淋巴瘤鉴别的关键点。但是多数WM病理结果并不典型，较难区分，给WM病理诊断带来困难。此外，即使存在较为明显的小梁间隙侵犯，而无其他WM典型表现时，也不能直接诊断WM[7]。

4. 免疫表型无特征性标志。WM目前并无特征性的免疫表型，常见的免疫表型为CD19（+），CD20（+），sIgM（+），CD5（−），CD10（−），CD22（+），CD23（−），CD25（+），CD27（+），FMC7（+），通常CD38和（或）CD138（+），而CD103（−）[5]。但是仍然有10％～20％的患者可表达CD5、CD10或CD23。仅凭免疫表型较难与其他惰性B淋巴细胞增殖性疾病鉴别，导致诊断困难。

5. MYD88^L265P^突变是WM常见突变类型[8]，但不是WM特异性突变[9]，还可见于其他B细胞淋巴瘤，如慢性淋巴细胞白血病（5％～10％）、边缘区淋巴瘤（5％～10％）、非生发中心型弥漫大B细胞淋巴瘤（30％～40％）等。另外，在IgM型意义未明的单克隆丙种球蛋白血症（MGUS）患者中，MYD88^L265P^突变高达50％～80％[6]。因此，即使MYD88^L265P^突变对于WM既不特异，也不必需，我们仍应综合进行鉴别诊断。

6. 临床表现差异明显。WM临床多以乏力、贫血为主要表现，其他临床特征还包括高黏滞血症、头晕、视物模糊、出凝血异常、神经系统症状等，使得患者常就诊于非血液科室，不能及时确诊[7]。

总体而言，LPL/WM无特异的形态学、免疫表型及遗传学改变，临床症状多样，故LPL/WM在诊断过程中存在诸多难点。对于存在单克隆IgM分泌及淋巴样浆细胞形态或同时存在单克隆B细胞和浆细胞时应怀疑WM，此后进一步结合患者骨髓活检、淋巴结、肝脾肿大及MYD88突变的情况进一步明确诊断。此外，WM诊断是一个排他性诊断，需要紧密结合临床表现及病理学等检查结果进行综合诊断。中国华氏巨球蛋白血症工作组经多次讨论，在2022版指南中初步制定了一套LPL/WM鉴别诊断流程（见指南）[5]，供临床工作参考。

三、WM与IgM型MGUS及IgM型多发性骨髓瘤（MM）如何鉴别

目前IgM型MGUS的诊断标准有两个，一个是IWWM标准[1]：①有血清单克隆IgM蛋白；②骨髓中无淋巴浆/浆细胞浸润；③无其他B淋巴细胞增殖性疾病的证据；④无相关器官或组织受损的证据，如淋巴瘤浸润所致贫血、肝脾肿大、高黏滞血症、系统性症状或淋巴结肿大，以及浆细胞疾病所致溶骨性损害、高钙血症、肾功能损害或贫血。另外一个是WHO 2016版诊断标准[2]：①有血清单克隆IgM蛋白，浓度<30 g/L；②骨髓中单克隆浆细胞占比<10％；③无其他B淋巴细胞增殖性疾病的证据；④无相关器官或组织受损的证据，如淋巴瘤浸润所致贫血、肝脾肿大、高黏滞血症、系统性症状或淋巴结肿大，以及浆细胞疾病所致溶骨性损害、高钙血症、肾功能损害或贫血。两个标准有不一致之处，WHO标准更强调IgM定量和骨髓侵犯负荷，WHO标准中MGUS与目前的WM标准存在重叠，如部分无症状的骨髓侵犯程度小于10％的WM也符合IgM型MGUS的诊断标准，这一点在WHO标准中已经特别指出。因此，在WM中国指南中采用了IWWM的IgM型MGUS标准。

WM与IgM型MM常需要鉴别。虽然IgM型MM具有典型的临床及病理学特征，如细胞形态学为浆细胞形态，免疫表型高表达CD38、CD138，不表达CD19、CD45，常伴溶骨性损害及肾功能不全等，但仍有极少数WM以浆细胞形态居多，且可伴有肾功能不全[10]，从而给诊断造成干扰。此外，虽然MM多不表达CD20[11]，且无淋巴结肿大，但是伴有t（11;14）的MM通常伴随CD20的表达，且部分患者也可出现淋巴结肿大，增加了诊断的难度。值得注意的是，MM常伴有14q32（IGH）易位，而并不伴MYD88^L265P^基因突变，这是两者重要的鉴别点。

四、如何进行MYD88/CXCR4基因突变检测

前期研究通过配对肿瘤和正常全基因组测序，发现在91％的WM患者中存在MYD88^L265P^突变[12]。随后多项研究通过对CD19阳性分选的WM肿瘤细胞进行Sanger测序或等位基因特异性聚合酶链反应（AS-PCR）检测发现WM患者MYD88^L265P^检出率达93％～97％[13]–[15]，而在未分选的样本中MYD88^L265P^检出率明显降低。此外，近些年随着更为敏感的微滴式数字PCR（ddPCR）的出现，可实现MYD88的绝对量化，对于不分选的肿瘤标本，前期数据显示MYD88的阳性检出率达87.5％左右[16]。陶怡等[17]综合比较了二代测序（NGS）、AS-PCR和Sanger法对MYD88的检出率，整体而言，未分选情况下，NGS和AS-PCR技术优于Sanger法。

CXCR4是WM第二常见的突变，使用NGS检测，突变率为30％～40％，靶点主要为CXCR4 C末端结构域的体细胞突变[18]。前期研究证实几乎所有CXCR4突变均伴MYD88突变，但与MYD88特异性突变位点不同，目前在WM患者中已检测出40多种CXCR4 C末端结构域的无意义和移码突变[19]。Treon等[19]使用Sanger测序在近30％的WM患者中检测出CXCR4突变，其中最常见的是CXCR4^C1013G^突变，约占所有CXCR4突变患者的30％，占WM的8％。因此，仅凭借AS-PCR和Sanger测序较难覆盖CXCR4所有突变位点，应该使用如NGS等更为精准的方法进行检测。

因此，NGS检测是MYD88及CXCR4的最佳检测方法，测序深度2 000 ×，敏感性可达1％，基本可满足临床需要，若能进行分选，检出率将进一步提高。若无条件进行NGS检测，AS-PCR或者ddPCR优于传统Sanger法。在样本选择上，骨髓或淋巴结的检出率明显高于外周血样本[17]。

五、无症状患者如何预测疾病进展

对于初诊无治疗指征患者，哪些因素可能预测疾病进展？美国哈佛大学曾分析了23年间439例无症状WM患者的转归，发现IgM定量≥45 g/L、骨髓浸润≥70％、β_2_-微球蛋白≥4.0 mg/dl、白蛋白<35 g/L是独立治疗指征预测因素，依据上述4个因素构建的线上模型（www.awmrisk.com）可将患者分为低/中/高危三组，中位至需要治疗时间分别为1.8年/4.8年/9.3年，该模型得到欧洲48例样本的验证，因此值得参考[20]。

六、如何选择治疗方案

（一）一线治疗方案选择

对于有治疗指征的WM患者，初始治疗方案的选择主要考虑以下因素：患者年龄与耐受性、合并症情况如是否合并外周神经炎、IgM定量、肿瘤负荷、MYD88^L265P^和CXCR4基因突变状态、患者的治疗意愿与经济能力、是否能坚持长期治疗以及各个方案的疗效等。

指南中优先推荐的5个方案中，除了泽布替尼与伊布替尼进行过头对头比较，其他均缺乏直接比较。来自美国梅奥诊所的回顾性研究和一项meta分析显示苯达莫司汀联合利妥昔单抗（BR）方案在深度缓解率和无进展生存（PFS）率上均优于利妥昔单抗联合环磷酰胺及地塞米松（RCD）和利妥昔单抗联合硼替佐米及地塞米松（BDR）方案[21]–[22]，但哈佛大学DFCI数据显示，三个化疗方案的中位PFS时间无显著差异，均为5年左右[23]。2022年ASCO年会上报道了年龄匹配的MYD88突变型WM接受BR方案治疗6个疗程与伊布替尼单药持续治疗的疗效，4年PFS率（72％对78％，*P*＝0.14）和OS率接近（95％对86％，*P*＝0.3）[24]。ASPEN研究直接比较了泽布替尼与伊布替尼治疗MYD88突变型WM的疗效，从非常好的部分缓解（VGPR）率上来看，泽布替尼高于伊布替尼（28％对19％，*P*＝0.09）[25]，不良反应相对较小，特别是房颤发生率低。美国梅奥诊所的数据显示，一线接受免疫化疗复发后接受BTK抑制剂组与一线接受BTK抑制剂复发后接受免疫化疗组的生存时间相当[26]。因此，单纯从疗效上来看，BTK抑制剂与BR、BDR、RCD方案无显著差异。

（二）复发难治患者的治疗选择

WM目前仍是不可治愈的疾病，多数患者最终复发进展，对于常规治疗后复发的患者仍应结合治疗指征及复发时间选择治疗方案。初始治疗三年以上复发的患者可继续原方案治疗，三年以内复发的患者可选择与既往治疗无交叉耐药的方案。由于自体造血干细胞移植存在一定的移植相关死亡率，因此目前不作为患者复发后的常规选择方案。其他新药如BCL2抑制剂、二代BTK抑制剂等均显示出良好的疗效，是复发患者重要的治疗选择。

（三）维持治疗选择

由于缺少前瞻性的临床数据，目前是否进行维持治疗仍存在争议。既往研究显示使用利妥昔单抗联合治疗的患者应用利妥昔单抗维持治疗，41.8％的患者疗效进一步提高，且PFS及OS时间均较未维持治疗组明显延长[27]。但该研究是回顾性研究，包括初诊和既往接受治疗的患者，且前期诱导治疗方案多样，存在不可预见的偏倚。此外，StiL2008研究显示，使用BR方案诱导治疗达到部分缓解以上的患者，使用利妥昔单抗维持治疗生存获益不明显[28]。因此，2022版指南并未对维持治疗进行常规推荐。对于使用利妥昔单抗治疗后是否进行维持治疗，应该充分结合患者前期诱导治疗方案、治疗意愿、患者疗效及一般情况进行综合选择。且值得注意的是，使用利妥昔单抗维持治疗的患者发生感染的风险较未维持治疗组增加[27]，因此选择维持治疗时应充分考虑患者感染的风险。

七、WM疗效评价与治疗目标

（一）WM疗效评价

目前WM疗效评价标准仍遵循第六届国际华氏巨球蛋白血症工作组[29]相关推荐，结合临床症状、IgM定量、免疫固定电泳、骨髓及影像学变化等进行综合评估，具体评估请参照指南。但是值得注意的是，现有疗效评估仍然存在一定的难点。

1. VGPR定义中对髓外病灶的改变存在一定争议：虽然髓外病灶如肿大的淋巴结或脾脏在WM患者中比例并不高（<30％），但多数患者即使接受联合化疗也较难获得髓外病灶的完全缓解（CR）。且2013年第六届国际华氏巨球血症工作组及2018年ESMO指南[30]中将VGPR仍定义为血清IgM定量下降≥90％，原有的髓外病灶（如肿大的淋巴结或脾脏）消失，无新的疾病活动的症状或体征。但是目前NCCN指南[31]及多数临床研究[32]均未对VGPR患者的髓外病灶进行具体要求，NCCN指南定义的VGPR为血清IgM定量下降≥90％；体格检查或CT检查显示原有的髓外病灶缩小，如肿大的淋巴结或脾脏；无新的疾病活动的症状或体征。本版指南中我们采用了NCCN的改良疗效评价标准。

2. IgM定量的检测方法不统一：目前第六届国际华氏巨球蛋白血症工作组疗效评价标准并未对IgM的检测方法进行限定，无论通过密度法测定M蛋白定量还是通过比浊法检测总血清IgM均是可行的检测方式，且均与骨髓反应密切相关[33]。但通过比浊法评估总的IgM定量高于通过密度测定法测定的M蛋白值[34]。且有研究显示，在同一实验室中使用相同的方法对患者进行序贯反应评估，发现通过密度测定法定量M蛋白和通过比浊法定量免疫球蛋白的生物学变异性分别为8％和13％[35]。

此外，两种方法均存在一定的局限性[36]。当患者存在冷球蛋白血症时，无论是使用比浊法还是密度法检测，数值均不准确。对于初诊IgM较低的患者，当前的疗效评价体系也不能准确进行疗效评价，对于这部分患者应当结合患者骨髓活检及髓外情况进行综合评价。

另外，虽然比浊法测定总IgM较易进行自动化检测，但无法区分背景IgM和疾病相关IgM。且这种检测方法容易过度估计副蛋白，在血清IgM水平非常低或非常高的患者中，误差较大。而密度测定法主要是根据血清蛋白大小和净电荷对IgM型M蛋白进行分离，通过光学扫描生成电泳图，但M峰的边界界定主要依靠训练有素的技术人员手动描绘，存在一定的人为误差。此外，IgM丙种球蛋白在检测过程中可发生聚集而形成聚集体，无法通过电泳，从而影响检测结果。且通常IgM丙种球蛋白以可溶的五聚体或其他高阶复合物的形式存在，在电泳图上会产生多个重叠峰，给实验室评估带来一定的挑战性。最后，虽然多数免疫球蛋白位于γ区，但IgA和IgM更易在β区域聚集，而该区域包含大量正常的血清蛋白，从而影响定量的准确性。

血清游离轻链测定（sFLC）目前广泛应用于MM，并已证明对非分泌性和仅轻链疾病患者具有较好的应用价值。其在WM中研究较有限，部分研究显示其对治疗反应和疾病进展有早期提示作用[37]。但专家组认为目前没有足够的数据将sFLC和重轻链（HLC）评估纳入修订后的疗效评价标准，并鼓励进一步的前瞻性评估。

因此，对于WM患者如何进行IgM的检测仍无统一标准，但值得注意的是，对于接受系统治疗的患者，应当对IgM进行连续评估，检测患者IgM的动态变化，并结合患者治疗反应的变化及幅度进行综合判断。

3. IgM定量受治疗影响，疗效评估最佳时间不明确：目前多项研究均显示，WM患者IgM定量受治疗影响明显。有研究显示利妥昔单抗单药或联合化疗可能导致IgM水平升高并可能持续数月[38]，而硼替佐米可能会较短时间内抑制IgM分泌但不杀伤肿瘤细胞[39]。此外，不同治疗方案达到最佳治疗反应的时间并不一致，对于以利妥昔单抗为基础的方案，部分患者达最佳疗效的时间达6个月。且停止治疗后仍有部分患者可持续获益。因此，对于这部分患者，更应该密切观察患者的临床表现、血常规及影像学变化等进行综合评估，必要时进行骨髓活检评估患者肿瘤负荷变化等。

4. 骨髓微小残留病（MRD）检测对预后的评价并不明确：在当前疗效评估体系中仅建议对考虑获得CR的患者进行骨髓流式细胞术及活组织检查等相关检测，骨髓MRD监测对预后的判断价值并不明确。且完成治疗后评估骨髓反应的最佳时间点也是未知的，可能因所给予的治疗类型不同而异。

（二）治疗目标

WM是一种惰性淋巴瘤，其首要治疗目标是症状缓解，而不是缓解深度。与其他淋巴瘤不同，目前治疗方案下大多数WM患者不能达到CR，因此，对于治疗有效的患者完成既定疗程（通常为6个疗程）或达到疾病平台期后即可结束治疗或进入维持治疗，并不需要追求更深度的缓解（如VGPR及CR）。此外，WM起效相对缓慢，且通常临床症状如贫血的改善早于肿瘤负荷的降低，如无确切疾病进展证据，不宜频繁更换治疗方案。

与2016版专家共识相比，本版指南在诊断方面提供了临床诊断思维流程，在治疗选择上也提供了流程图，便于临床参考。鉴于LPL/WM的临床罕见性，无论是疾病本质的研究还是治疗选择的循证医学证据水平都有待进一步提高，因此，我们更期待国内同行携手，努力提高我国LPL/WM诊治与研究水平，造福患者。
